# Supplementary material for: Superfluid motion and drag-force cancellation in a fluid of light
Source: Nat Commun. 2018 May 29;9:2108. doi: 10.1038/s41467-018-04534-9 (PMC5974130; doi:10.1038/s41467-018-04534-9)
Supplement: Supplementary file 1 — Supplementary Information [file 41467_2018_4534_MOESM1_ESM.pdf]

Supplementary information
Superfluid motion and drag-force cancellation in a fluid of light

Michel et al

SUPPLEMENTARY NOTE 1 — Expression of the fluid velocity

In Eq. (1) of the main text, the gradient of the phase of the complex envelope E_f of the electric field $\mathrm{Re}(E_\mathrm{f}\, e^{i n_\mathrm{e} k_\mathrm{f} z})$ play the role of the velocity $v = (n_\mathrm{e}\, k_\mathrm{f})^{-1}\, |\partial \arg(E_\mathrm{f})/\partial \mathbf{r}|$ of the fluid of light. In the experiment, the fluid-of-light beam consists in a gaussian beam which is large compared to the size of the obstacle (see Figure 1a). It can be approximated by a plane wave, such as $E_\mathrm{f} \propto e^{i\mathbf{k}_\perp \cdot \mathbf{r}}$, with $k_\perp = k_\mathrm{f} \sin\theta_\mathrm{in}$ the transverse wave vector of the plane wave. Its phase $\mathbf{k}_\perp \cdot \mathbf{r}$ thus remains constant in the vicinity of the obstacle. Consequently, v is only given by θ_in, the angle between the fluid-of-light beam and the z direction: $v = \sin\theta_\mathrm{in}/n_\mathrm{e} \simeq \theta_\mathrm{in}/n_\mathrm{e}$ in the here-considered paraxial approximation.

SUPPLEMENTARY NOTE 2 — Dispersion relation and healing length

Considering the Bogoliubov theory of weak perturbations on top of a uniform fluid of light, the dispersion relation reads [1, 2]

$$\mathcal{W}(k_\perp) = \sqrt{\frac{k_\perp^2}{2n_\mathrm{e}k_\mathrm{f}}\left(\frac{k_\perp^2}{2n_\mathrm{e}k_\mathrm{f}} + k_\mathrm{f}\Delta n(I_\mathrm{f})\right)} \tag{1}$$

The quantity $\xi = [n_\mathrm{e}\, k_\mathrm{f} \times k_\mathrm{f}\, |\Delta n(I_\mathrm{f})|]^{-1/2}$ is called the healing length and can thus be extracted from the previous equation. It defines a length scale for the smallest intensity modulations that can occur in the system. In the main text, the size of the obstacle is compared to the healing length of the fluid of light. Nevertheless, this quantity is intensity-dependent in the here-considered case. The calculated values are plotted in Supplementary Figure 1. The red dashed line corresponds to the radius of the obstacle (estimated experimentally at 6 μm).

SUPPLEMENTARY NOTE 3 — Optical analog of the Ehrenfest relations

The Ehrenfest theorem relates the time derivative of the expectation values of the position and momentum operators x and p to the expectation value of the force $F = -dV/dx$ on a massive particle moving in a scalar potential,

$$m\frac{d}{dt}\langle x\rangle = \langle p\rangle,\ \frac{d}{dt}\langle p\rangle = -\left\langle \frac{\partial V(x)}{\partial x}\right\rangle \tag{2}$$

while the equation describing the motion of a massive particle in a potential is the Schrödinger equation $i\hbar\partial_t\psi = -\frac{\hbar^2}{2m}\Delta\psi + V(x,t)\psi$ and the Hamiltonian reads $H(x,p,t) = \frac{p^2}{2m} + V(x,t)$. Using

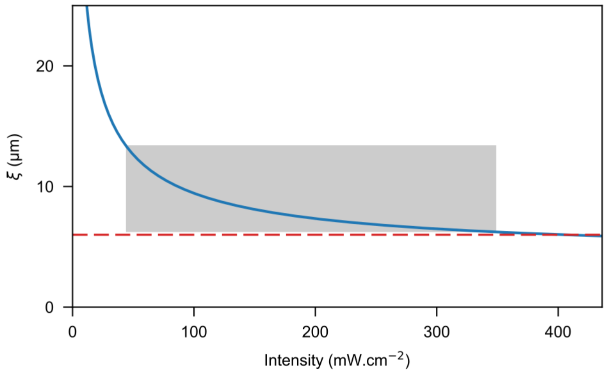

Supplementary Figure 1: **Theoretical variation of the healing length ξ with respect to the intensity**. The dashed red line corresponds to the radius of the obstacle. The gray box defines the experimentally accessible range.

the analogy between the Schrödinger equation and the propagation equation for the obstacle beam in the optical potential $\Delta n(I_{\mathrm{f}}) + \Delta n(I_{\mathrm{ob}})$ which reads

$$i\partial_z E_{\mathrm{ob}} = -\frac{1}{2n_{\mathrm{e}}k_{\mathrm{ob}}}\nabla^2 E_{\mathrm{ob}} - k_{\mathrm{ob}}\left[\Delta n(I_{\mathrm{f}}) + \Delta n(I_{\mathrm{ob}})\right] E_{\mathrm{ob}} \tag{3}$$

we can write the Hamiltonian which takes the form

$$H(x, k_\perp, z) = \frac{k_\perp^2}{2n_{\mathrm{e}}k_{\mathrm{ob}}} - k_{\mathrm{ob}}\left[\Delta n(I_{\mathrm{f}}) + \Delta n(I_{\mathrm{ob}})\right] \tag{4}$$

Denoting $\langle x\rangle = \int x|E_{\mathrm{ob}}|^2\, dx$ the position of the centroid of the obstacle beam, the optical analog of the Ehrenfest theorem reads:

$$(n_{\mathrm{e}}k_{\mathrm{ob}})\frac{d}{dz}\langle x\rangle = \langle k_\perp\rangle \tag{5}$$

$$\frac{d}{dz}\langle k_\perp\rangle = -\left\langle -k_{\mathrm{ob}}\frac{\partial\left[\Delta n(I_{\mathrm{f}}) + \Delta n(I_{\mathrm{ob}})\right]}{\partial x}\right\rangle \tag{6}$$

which leads to the equation of motion for the centroid of the obstacle beam:

$$(n_{\mathrm{e}}\, k_{\mathrm{ob}})\,\partial_{zz}\langle x\rangle = \partial_x[k_{\mathrm{ob}}\,\left[\Delta n(I_{\mathrm{f}}) + \Delta n(I_{\mathrm{ob}})\right]] \tag{7}$$

It is worth mentioning that the influence of the Self-Phase Modulation on the obstacle beam shape is actually negligible with respect to the displacement induced by the fluid of light. Eq. 7 thus simplifies to :

$$(n_{\mathrm{e}}\, k_{\mathrm{ob}})\,\partial_{zz}\langle x\rangle = \partial_x[k_{\mathrm{ob}}\,\Delta n(I_{\mathrm{f}})] \tag{8}$$

Obstacle displacement in the fluid of light at rest.— We consider here that the obstacle beam propagates linearly, so that it does not induce any refractive index modification. In this case, the fluid of light behaves as a "stationary" gaussian potential in which the obstacle beam can move in the transverse direction with respect to its initial position. By assuming that $\Delta n(I_{\mathrm{f}})$ is z independent,

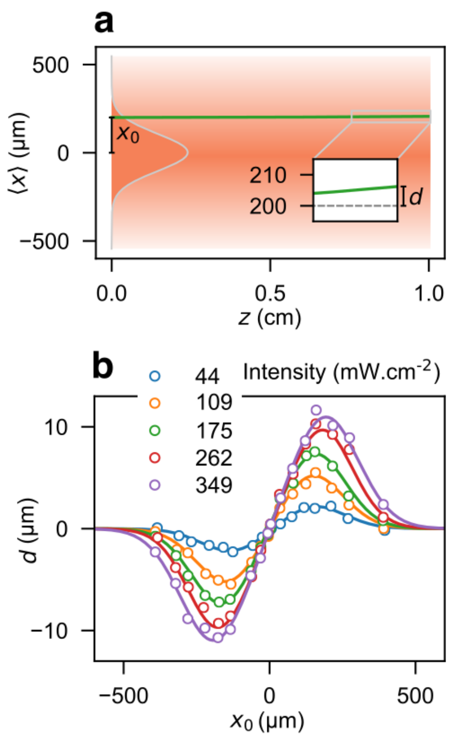

Supplementary Figure 2: **Transverse displacement of the obstacle in a gaussian potential.** (**a**) Calculated transverse displacement $\langle x \rangle$ along the propagation, z, axis for a potential induced by a 270 μm at $1/e^2$ half-width gaussian laser beam of intensity $I_\mathrm{f} = 175$ mW.cm^{-2}. $x_0 = 200$ μm is the initial position of the obstacle. d is the transverse displacement, with respect to x_0, at the crystal's output. (**b**) Measured transverse displacement for various laser beam intensities I_f ranging from 44 to 349 mW.cm^{-2} as a function of x_0. The fit procedure (solid lines) allows to extracted $I_\mathrm{sat} = 380 \pm 50$ W.cm^{-2} and $\Delta n_\mathrm{max} = 2.5 \pm 0.4 \times 10^{-4}$.

which is valid in the here-considered linear propagation of the obstacle beam, we readily obtain from eq. 8

$$d = \langle x(z) \rangle - x_0 = \frac{1}{2} \left[\partial_x \Delta n(I_\mathrm{f})/n_\mathrm{e} \right] z^2 \tag{9}$$

where x_0 is the initial position of the obstacle.
As shown in Supplementary Figure 2a, for $x_0 = 200$ μm and an optical potential induced by a 270 μm wide gaussian beam of intensity $I_\mathrm{f} = 175$ mW.cm^{-2}, the relative transverse displacement d defined in eq. 9 reaches 7.8 μm at the output of the crystal (see inset). The experimental measurement of d, for various intensities and positions x_0, is presented in Supplementary Figure 2b. The experimental data are fitted (solid lines), using the above expression, the saturation intensity and the maximum refractive index modification being the fitting parameters. We extract $I_\mathrm{sat} = 380 \pm 50$ mW.cm^{-2} and $\Delta n_\mathrm{max} = 2.5 \pm 0.4 \times 10^{-4}$. It is worth mentioning that the value of I_sat is used for the calculation of $\Delta n(I)$ and its deriving quantities (i.e., c_s and ξ).

Obstacle displacement in a moving fluid of light.— When one performs the experiment in the moving

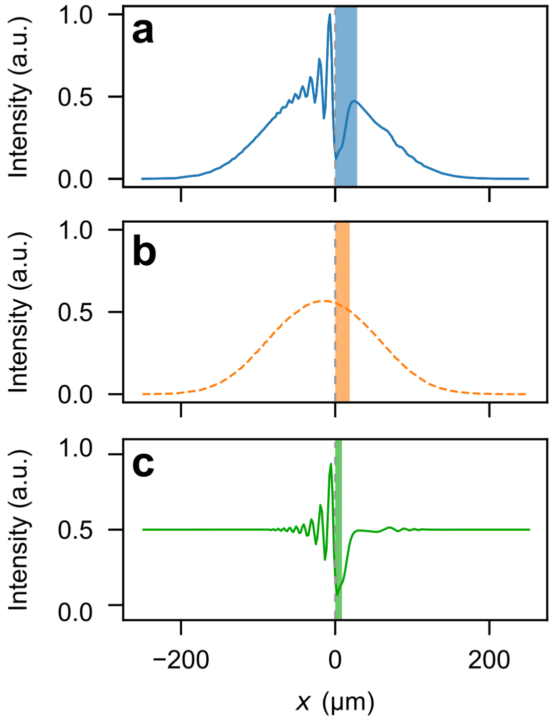

Supplementary Figure 3: **Illustration of the measurement of the net obstacle displacement.** In each panel, the profile corresponds to a cut along the x axis of the spatial distribution of intensity. The dashed grey line corresponds to the all-optical obstacle position. (**a**) Typical profile of the fluid of light intensity. The total corresponding transverse displacement is illustrated by the blue region. (**b**) Typical profile of the unperturbed fluid of light intensity (i.e. when the obstacle is weak). The transverse displacement induced by the gaussian shape of the fluid of light is depicted by the orange region. (**c**) The net transverse displacement of the obstacle induced by the local intensity modulation (green curve) is shown by the green region.

fluid of light case to probe the transition to superfluidity, two effects cause the displacement of the obstacle beam. The first one is the relative initial position of the obstacle beam with respect to the gaussian shape of the fluid of light beam. The second is due to the asymmetry of the fluid of light beam. The latter is the displacement associated to the drag force, but is much smaller than the first one. The measurement described in the previous paragraph allows one to extract the net displacement of the obstacle beam, which is exclusively conditioned by the asymmetry of the fluid of light beam. In Fig. 3f-j of the main text, the net transverse displacement of the obstacle induced by the local modulation of the fluid of light intensity is plotted for various initial conditions. As seen previously, the all-optical obstacle is highly sensitive to the surrounding refractive index potential resulting essentially from the gaussian distribution of the fluid of light intensity. Thus, for each data point, we subtract the displacement measured at very low obstacle intensity, when its influence on the fluid of light is negligible. This correction procedure is illustrated in Supplementary Figure 3. Supplementary Figure 3a represents a cut along the x-axis of a spatial distribution of intensity in the supersonic case. The blue box represents the displacement of the obstacle between the input and

the output of the crystal. Supplementary Figure 3b shows the displacement of the obstacle for the same initial condition but without any influence of the obstacle on the fluid of light. Supplementary Figure 3c depicts the net displacement obtained from Supplementary Figure 3a and corrected by subtracting the intrinsic effect of the gaussian shape of the fluid-of-light beam (Supplementary Figure 3b).

SUPPLEMENTARY NOTE 4 — Negative velocities measurement artifacts

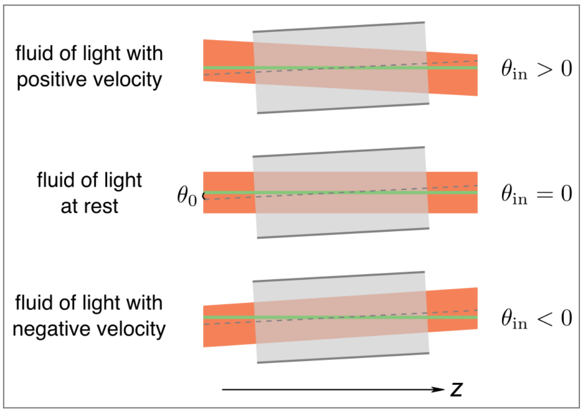

Supplementary Figure 4: **Experimental configurations.** To avoid cavity effects, the crystal is slightly tilted with an angle θ_0. For positive fluid of light velocities (top), no interferences patterns are expected. For negative velocities (bottom), as $\theta_{\rm in}$ approaches θ_0, large interferences pattern appear.

To avoid typical cavity effects (large interferences appearing when the light propagates perpendicularly to the faces of the sample), the crystal was slightly tilted with an angle θ_0 with respect to the propagation direction. This situation is illustrated in Supplementary Figure 4. The positive values of the fluid of light velocity are defined for input angles $\theta_{\rm in} > 0$ (Supplementary Figure 4, top). In this case, no interference effects are expected. However, for negative velocities defined for $\theta_{\rm in} < 0$ (Supplementary Figure 4, bottom), as $\theta_{\rm in}$ approaches θ_0, large interferences pattern appear. Typical images of the output intensity, shown in Supplementary Figure 5a, exhibit such patterns (pointed out by the red arrows). Note that this set of data can not be compared directly with the figure 2 of the manuscript since the experimental conditions are different (mainly, the acquisitions were performed with a 4× imaging microscope objective instead of a 20× in the manuscript). Qualitatively, the images look very similar with the disappearance of the diffraction pattern as v/c_s tends to zero. The associated obstacle displacements, illustrated in Supplementary Figure 5b, also present similarities with an increase (resp. decrease) at low (resp. high) Mach number and comparable values (about 1.5 μm maximum). However, as shown in Supplementary Figure 5c which represents the displacement along the y axis expected to be zero, the measurement's uncertainty is much larger (at least twice) than for the results presented in the manuscript. In these conditions, the cavity effects blur the signal and forbid any serious quantitative analysis. It thus becomes tough to extract sensitive quantities such as the transition threshold and the plateau. This is the reason why we focused our study on positive values of the fluid velocity only. We presented the results for a very small interval of negative velocities (actually not blurred by the large interferences) to show the tendency for the obstacle to move towards the opposite direction.

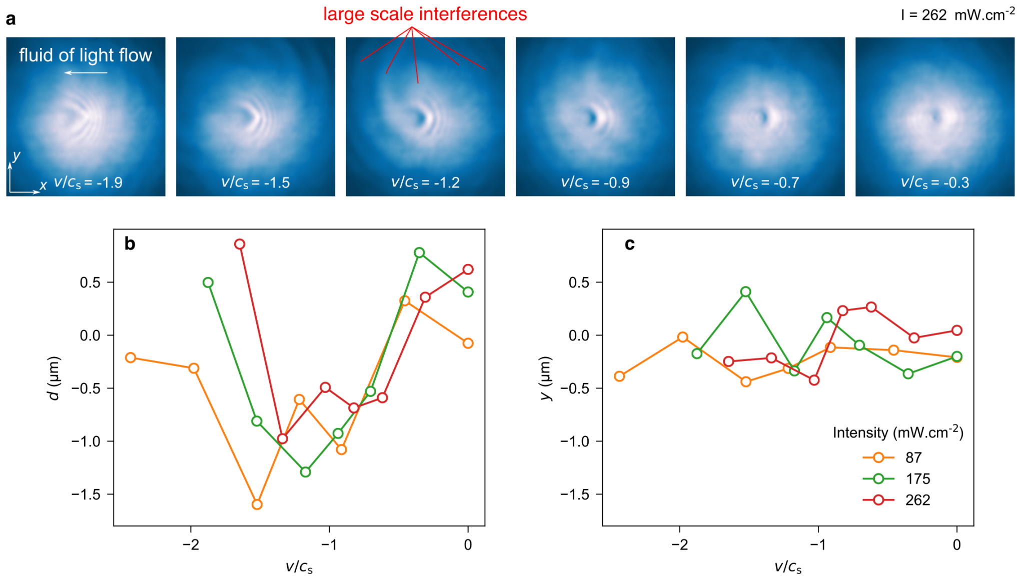

Supplementary Figure 5: **Illustration of the cavity effects appearing at negative v/c_s.** (**a**) Spatial distribution of the output intensity of the fluid of light for various negative v/c_s at $I = 262$ mW.cm^{-2}. The red arrows denote large scale interferences. (**b**) Measurement of the transverse displacement of the obstacle induced by the local modulation of the intensity of the fluid of light for various input conditions. (**c**) Measurement of the transverse displacement of the obstacle along the y axis, which is expected to be zero. This defines the typical uncertainty in the measured displacements.

Note that these blurring cavity effects are inherent to any propagating geometries. One solution would has been to cut and polish, at a small angle, one face of the crystal.

SUPPLEMENTARY NOTE 5 — Qualitative discussion on the obstacle extra displacement

In Fig. 3f-j of the main text, we observe that the displacement is not purely zero in the superfluid regime for large intensities. We claim that this is likely due to the displacement acquired during the non-stationary regime at the early stage of the propagation. Here, by means of numerical simulations giving access to the evolution along z of the fluid of light field, we qualitatively discuss this argument. The images in Supplementary Figure 6 shows numerical simulation of the evolution of the fluid of light obtained for $\theta_{\rm in} = 5$ mrad and (a) $I_{\rm f} = 44$ mW.cm^{-2}, i.e. $v/c_{\rm s} = 0.7$ and (b) $I_{\rm f} = 349$ mW.cm^{-2}, i.e. $v/c_{\rm s} = 0.3$. Each panel corresponds to the snapshots of the fluid of light intensity taken at various distances z ranging from 1 to 10 mm. The image size is reduced to 50×50 μm^2 to get focused on the intensity distribution in the vicinity of the obstacle. At large intensity, according to the symmetric intensity distribution observed for $z = 10$ mm (blue line panel Supplementary Figure 6b), no displacement is expected. However, the transient regime shows an asymmetric intensity distribution in the vicinity of the obstacle (dashed line panel Supplementary Figure 6b). The associated refractive index modification leads to an accumulated transverse displacement that remains when no force any longer applies on the obstacle.

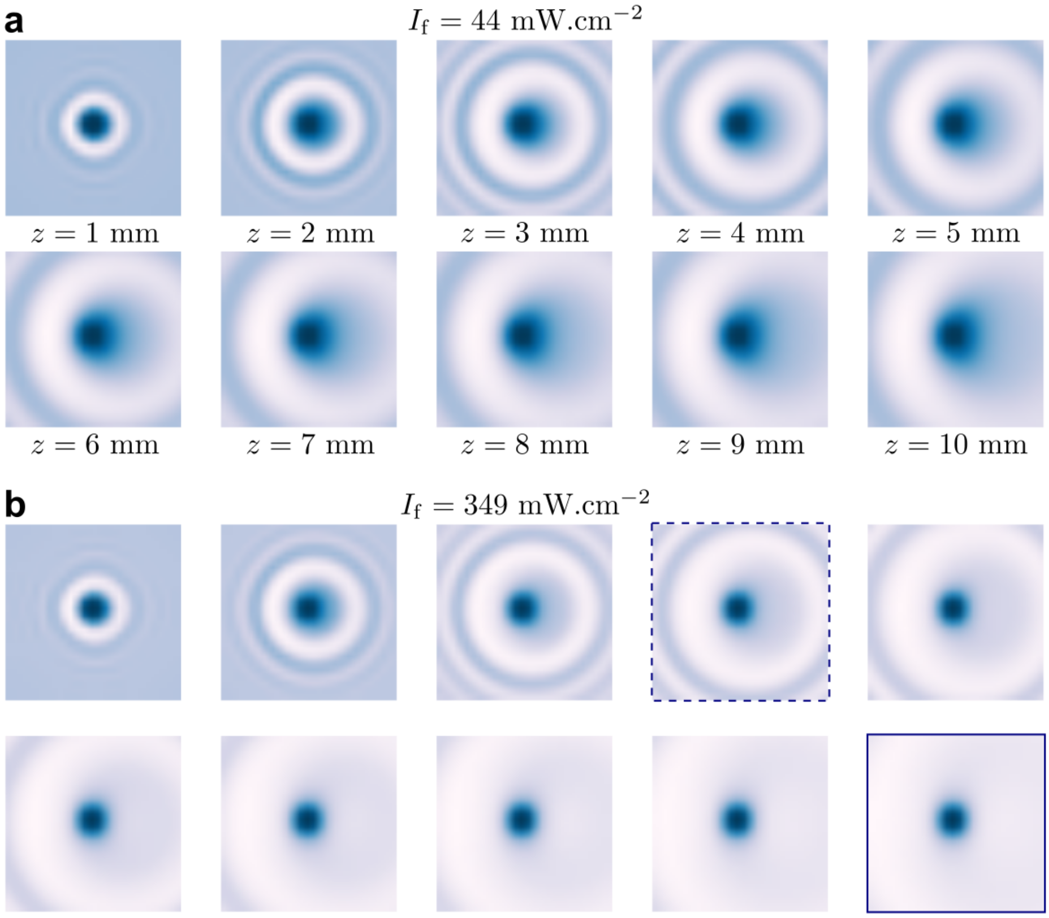

Supplementary Figure 6: **Numerical simulations of the evolution of the fluid of light**. In each panel, the images correspond to the snapshots of the fluid of light intensity taken at various distances z ranging from 1 to 10 mm for two different input intensities. (a) $I_f = 44$ mW.cm^{-2} and (b) $I_f = 349$ mW.cm^{-2}. The input angle is fixed at $\theta_{in} = 5$ mrad. The corresponding Mach numbers are respectively 0.7 and 0.3. The image size is 50×50 μm^2. The center of the images correspond to the position of the obstacle.

Supplementary References

[1] Carusotto, I. & Rousseaux, G. *The Cerenkov Effect Revisited: From Swimming Ducks to Zero Modes in Gravitational Analogues*, 109 – 144 (Springer International Publishing, 2013).

[2] Carusotto, I. Superfluid light in bulk nonlinear media. *Proc. R. Soc. A* **470**, 0320 (2014).
